# Comparison of Agonist Activity between CB1 and CB2 Receptors with Orthosteric Site Mutations

**DOI:** 10.3390/receptors3030018

**Published:** 2024-08-06

**Authors:** Christina A. Brust, Matthew A. Swanson, Christos Iliopoulos Tsoutsouvas, Snezana T. Dimova, Vuong Q. Dang, Edward L. Stahl, Jo-Hao Ho, Spyros P. Nikas, Alexandros Makriyannis, Laura M. Bohn

**Affiliations:** 1 The Herbert Wertheim UF Scripps Institute for Biomedical Innovation and Technology, Department of Molecular Medicine, Jupiter, FL 33458, USA; 2 The Skaggs Graduate School of Chemical and Biological Sciences at Scripps Research, La Jolla, CA 92037, USA; 3 Center for Drug Discovery and Department of Pharmaceutical Sciences, Northeastern University, Boston, MA 02115, USA; 4 Department of Chemistry and Chemical Biology, Northeastern University, Boston, MA 02115, USA

**Keywords:** G protein-coupled receptor, cannabinoids, internalization, AM12033, CP55,940

## Abstract

Human endocannabinoid signaling is primarily mediated by the cannabinoid receptors, CB1 and CB2, which are G protein-coupled receptors (GPCRs). These receptors have been linked to a variety of physiological processes and are being pursued as prospective drug targets due to their potential in treating pain and inflammation. However, because of their homology and shared signaling mechanisms, investigating the individual physiological roles of these receptors and designing subtype-selective ligands has been challenging. Using active-state CB1 and CB2 structures as guides, homologous residues within the orthosteric pocket of each receptor were mutated to alanine to test whether they equally impair CB1 and CB2 activity in response to two high-affinity, nonselective agonists (CP55,940 and AM12033). Interestingly, mutating the Y5.39 position impairs CB1 but not CB2 function. Conversely, mutating residue C6.47 improves CB1 but impairs CB2 signaling. TheF7.35A mutation leads to a decrease in CP55,940 potency at CB1 and impairs internalization; however, AM12033 gains potency and promotes CB1 internalization. In CB2, mutation of F7.35A decreases the potency of CP55,940 and neither agonist induces internalization. These observations provide some insight into functional sensitivity of CB1 and CB2 to different agonists when conserved residues are mutated in the orthosteric pocket.

## 1. Introduction

The endocannabinoid system facilitates a wide range of physiological processes, such as anxiety, depression, pain, inflammation, appetite regulation, learning and memory, thermogenesis, digestion, and neuronal development [1,2]. Endocannabinoids primarily interact with the G protein-coupled receptors (GPCRs) CB1 and CB2 which activate inhibitory G proteins (G_αi_) and inhibit adenylyl cyclase, thereby decreasing cellular cAMP levels [3–5]. The CB1 receptor is one of the most abundantly expressed GPCRs in the brain, largely found at the presynaptic terminals of inhibitory neurons [6]. It is also present in peripheral organs such as the liver and kidneys [7–9]. CB2 receptors are also present in the central nervous system, albeit to a lesser extent than CB1 [10,11]. CB2 is predominately expressed in immune cells [12,13] and is upregulated under certain pathological conditions [14,15]. Modulating CB2 activity has been proposed as a means of treating inflammatory disorders while avoiding neurological side effects associated with CB1 modulation. Additionally there are a number of indications where it may be desirable to selectively activate CB2while antagonizing or avoiding CB1 activation; these include chronic liver disease [16], obesity [17], binge-eating disorder [18], diabetes [19], and diabetic neuropathy [9,20,21].

CB1 and CB2 share 68% homology within the transmembrane domains [22], where most ligands interact. Therefore, it is not surprising that the majority of known cannabinoids display an affinity for both CB1 and CB2; furthermore, both of these receptors can activate G_αi_ [4,5]. In efforts to target immune function while avoiding psychoactive effects, some approaches have focused on developing CB2-selective agonists [23–25]. Due to the nonselectivity of most ligands and the conserved signaling mechanisms, studying the physiological contributions of each receptor in isolation is challenging. Recent advances in structural biology have revealed several high-resolution structures of CB1 and CB2, revolutionizing the approach to structure-guided drug design. The active-state CB1 receptor structure has been resolved with a number of ligands, including, (−)-7′-isothiocyanato-11-hydroxy-1′,1′ dimethylheptylhexahydrocannabinol (AM841) (PDB: 6KPG) [26]. AM841 is a (−)-*trans*- Δ^9^-tetrahydrocannabinol (Δ^9^-THC) derivative, the phytocannabinoid responsible for the psychotropic effects of *Cannabis sativa* [27]. However, unlike Δ^9^-THC, AM841 has an isothiocyanate (NCS) substituent, developed to covalently bind within the orthosteric binding site. The first active-state CB2 receptor structure was resolved with agonist AM12033 (PDB: 6KPF), which shares the same backbone as AM841 but contains a cyano moiety in place of the isothiocyanate and a shorter aliphatic chain [28]. Similarly to AM841, AM12033 was designed to irreversibly bind to the receptor, and it is likely that these modifications to the aliphatic tail facilitated the stabilization and capture of the CB1 and CB2 receptor structures [26,28].

While these structures are a valuable resource, they represent a static snapshot of how a ligand and modified receptors are captured in a nonbiological context. Even with emerging structures bound by more selective compounds, it remains difficult to discern what makes them selective, as many agonists orient within the orthosteric pocket comparably to nonselective agonists [29]. However, these structures inform us as to what amino acid residues may come in contact with an agonist and, by using site-directed mutagenesis, we investigated which residues at the ligand-binding interface influence agonist activity and if those residues differ between CB1 and CB2. To achieve this goal, we generated alanine mutations in eight orthogonal residues in each receptor and compared the G protein signaling activity generated by two high-affinity, selective agonists: AM12033 and CP55,940. A conserved mutation between CB1 and CB2 is expected to exhibit similar impairments in agonist activity if the residues are functionally homologous, although we recognize that individual mutations may have a far greater impact than directly affecting ligand interactions as activation barriers may be altered by subtle changes imparted by changes in seemingly benign residues. Moreover, we may miss important residues that were not predicted from solved active-state structures, such as those that impact on the ensemble of the orthosteric pocket and allosteric interactions [30–33]. Nonetheless, by utilizing functional assays and comparing the effects of two agonists, we find evidence that some conserved residues are essential for the function of both receptors while some impact one receptor more than the other.

## 2. Materials and Methods

### Molecular Docking.

Structural images and docking poses were generated using UCSF Chimera production version 1.17.3 [34] and Autodock Vina 1.2.0 [35,36]. Prior to docking, the solvent was removed, hydrogens were added, and any incomplete side chains were replaced using the Dunbrack 2010 rotamer library. [37]. The search volume was set to the orthosteric region of the receptor. Ligands CP55,940 and AM12033 were each docked individually into the CB1-G protein complex (PDB: 6KPG), and CP55,940 was docked into the CB2-G protein complex (PDB: 6KPF). The docking poses selected exhibited the lowest docking score and were determined to be the most energetically favorable.

### Chemicals.

CP55,940 was purchased from Sigma Aldrich (Product No: C1112, St. Louis, MO, USA). AM12033 was synthesized by the Makriyannis laboratory as previously described [28]. For the inhibition of cAMP stimulation, forskolin and 4-(3-Butoxy-4-methoxybenzyl)imidazolidin-2-one (Ro-20–1724) were purchased from Sigma Aldrich (Product Nos: F6886 and 557502, St. Louis, MO, USA). For live cell confocal imaging, anti-HA Dylight 488 conjugate was purchased from ThermoFisher Scientific (Product No: 26183-D488, Waltham, MA, USA). SR141716A used for overnight treatment was purchased from Tocris Bioscience (Product No: 0923, Minneapolis, MN, USA).

### Mutant CB1 and CB2 CHO Cell Line Generation and Maintenance.

Human CB1 and CB2 Chinese hamster ovary (CHO-K1) cells were generated as previously described [38]. The N-terminal 3xHA (haemagglutin) tagged receptor cDNA was purchased from cDNA.org (Bloomsburg, PA, USA) and subcloned into a murine stem cell retroviral vector (pMSCV-puro, Takara Bio USA, Inc., San Jose, CA, USA) using a Q5 site-mutagenesis kit (New England Biolabs, Ipswich, MA, USA). Mutant receptors were developed as previously described [26,38,39]; forward and reverse primers for mutagenesis were purchased from Sigma Aldrich and are provided in Supplemental Table S1. CB2 I110^3.29^A, S285^7.39^A, and F281^7.35^A constructs were ordered from GeneScript (Piscataway, NJ, USA). The retrovirus was packaged by Phoenix-Amphotropic Cells and applied to CHO-K1 cells for transduction. Expressing cells were grown under puromycin (Life Technologies, Carlsbad, CA, USA) selection (5 mg/mL). Single-cell isolation was used to ensure a homogeneous population of expressing cells via collection by fluorescence-assisted cell sorting (FACS) using a BD FACSAria^™^ III Cell Sorter (BD Biosciences, East Rutherford, NJ, USA) with an anti-HA DyLight^™^488 conjugate (1:200, ThermoFisher Scientific). Cells were maintained at 37 °C and 5% CO_2_ in complete media (1:1 DMEM/F12 (Life Technologies, Carlsbad, CA, USA) supplemented with 10% heat-inactivated fetal bovine serum (HI-FBS), 1% penicillin/streptomycin) supplemented with 5 mg/mL puromycin.

### Confocal Microscopy.

To validate the cell surface receptor expression in clonal populations after clonal selection, cells were plated onto 35 mm plates (MatTek Corporation, Ashland, MA, USA) coated with bovine collagen I (VWR, West Chester, PA, USA) and incubated overnight at 37 °C and 5% CO_2_ in complete media supplemented with 5 mg/mL puromycin. Live cells were stained with anti-HA DyLight^™^ 488 conjugate for 10 min at room temperature and then washed with PBS; cells were imaged in serum-free Opti-MEM (Life Technologies, Carlsbad, CA, USA).

For additional CB1 Y275A^5.39^ live cell imaging, cells were plated on collagen-coated 35 mm plates in either the presence or absence of 10 μM SR141716A with a final 0.1% DMSO in complete media and incubated overnight at 37 °C prior to imaging. Cells were serum starved for 30 min at 37 °C before imaging. For permeabilization, CB1 Y275A^5.39^ cells were fixed using PBS supplemented with 4% paraformaldehyde and 120 nM sucrose, then incubated for 10 min at room temperature. Next, cells were permeabilized with 0.25% Triton X-100 for 10 min at room temperature and rinsed with PBS. Then, cells were stained with the DyLight 488 HA-antibody (1:200) for 10 min at room temperature and rinsed with PBS prior to imaging in serum-free Opti-MEM.

For the F7.35A (CB1 F379A and CB2 F281A) experiments, cells were plated on collagen-coated 35 mm plates overnight in complete media. Then, cells were serum starved in serum-free, phenol-red-free MEM (Fisher Scientific, Hampton, NH, USA) for 30 min at 37 °C. Next, cells were stained with anti-HA DyLight^™^ 488 conjugate (1:300) for 10 min at room temperature, then baseline images to validate receptor expression were taken in serum-free, phenol red-free MEM. After baseline imaging, the vehicle or 10 μM drug was added (final 0.1% DMSO). Cells were maintained at room temperature and imaged at 30, 60, and 90 min, as indicated.

Microscopy was performed utilizing an Olympus Fluoview FV3000 (Center Valley, PA, USA) scanning confocal microscope with FV31S-SW software. Images were acquired with the objective and zoom indicated in the figure legend. Brightness and contrast were linearly adjusted for optimized presentation. Images were acquired with bright field white light in addition to fluorescence.

### cAMP Accumulation Assay.

Inhibition of forskolin-stimulated cAMP was performed using a Revvity cAMP Homogenous Time-Resolved Fluorescence resonance energy transfer (FRET) (HTRF) HiRange assay according to manufacturer’s instructions (Revvity, Waltham, MA, USA). Cells were plated (4000 cells/well) in white-walled, low-volume 384 well plates (Greiner, Kremsmünster, Austria) in Opti-MEM supplemented with 1% HI-FBS and 1% penicillin/streptomycin for 3 h at 37 °C. The cells were cotreated with 25 μM Ro-20–1724, 20 μM forskolin, and CP55,940 or AM12033 for 30 min at 37 °C. Detection reagents were added and incubated at room temperature for 1 h in the dark. Fluorescence was measured at 665 nm/620 nm using a Biotek Synergy Neo multi-mode plate reader (Agilent Technologies, Santa Clara, CA, USA). For the CB1 Y275A^5.39^ studies, wildtype CB1 and CB1 Y275A^5.39^CHO-K1 cells were incubated in the presence or absence of 10 μM SR141716A overnight (final 0.1% DMSO) in complete media supplemented with puromycin and 1% HI-FBS prior to plating.

### Data Analysis and Statistical Methods.

Responses in concentration response curves for each cell line were first normalized to their response over the vehicle and then presented as the % maximum of WT response for each agonist. Analysis was performed using GraphPad Prism v. 10 (San Diego, CA, USA) fitting to a 3-parameter non-linear regression constraining to a fixed baseline of 0 (since all data were normalized to a baseline of 1 following fold over vehicle analysis). For CP55,940, many of the curves were right shifted in the mutant receptors, making it difficult to reliably derive the EC_50_ since the response did not plateau at the highest concentration tested. In these cases, the maximum (“top”) of CP55,940 was constrained to be shared with the 100% WT response. No constraint (other than a bottom = 0) was applied for AM12033 analysis as all of the curves were defined. Statistical comparisons were made between the replicates of each mutant and the replicates of the WT to determine differences in the LogEC_50_ values (and Emax where indicated) with Prism using an extra sum-of-squares F test with an alpha value of 0.05. The LogEC_50_ (presented as −1 × LogEC_50_ = pEC_50_) values are presented in the tables as derived from the analysis of the replicates (n ≥ 3 individual experiments performed in duplicate wells) with 95% confidence intervals. The graphs of the curves show the mean with S.E.M.

## 3. Results

**Figure 1.**
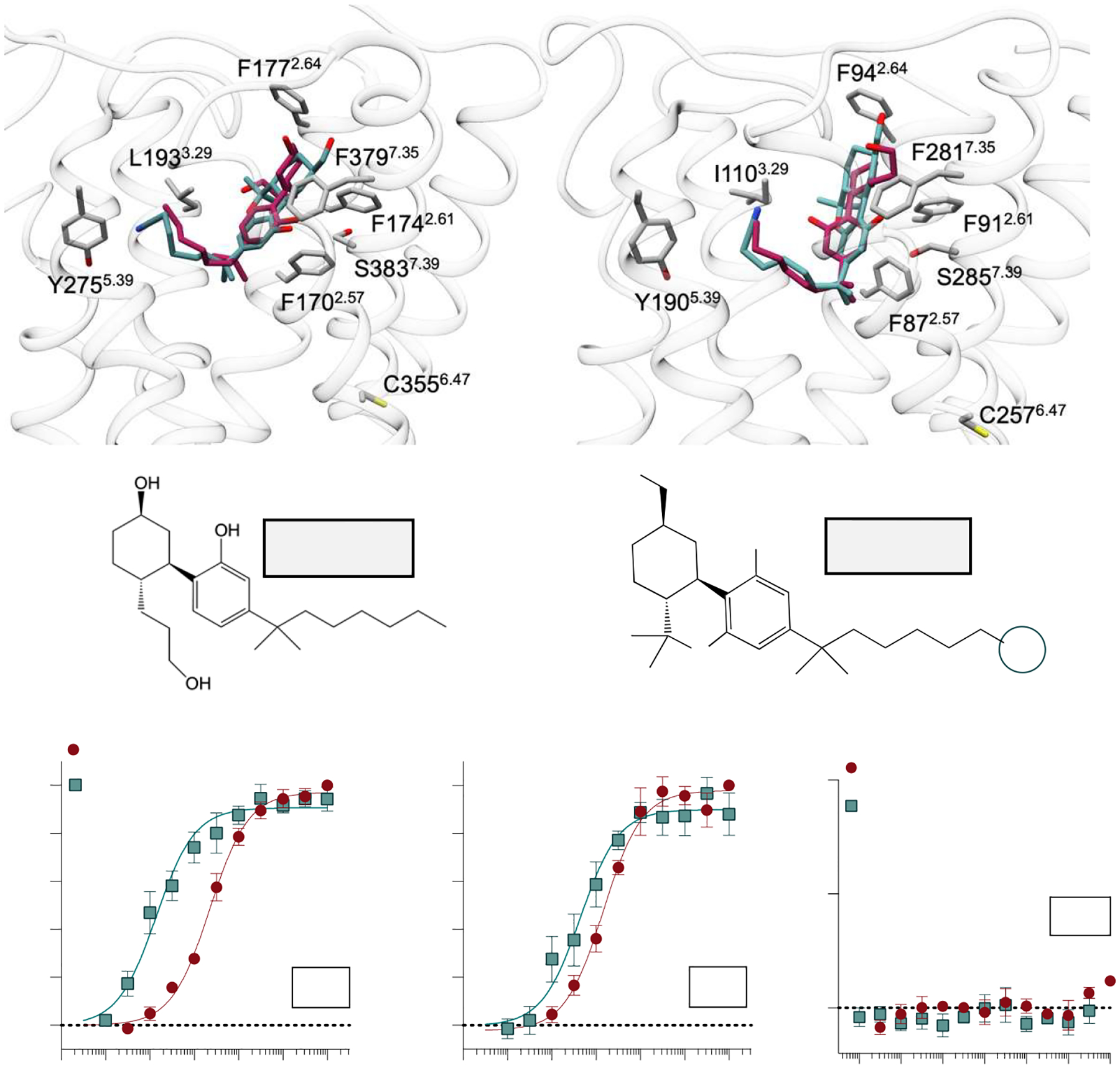
Characterization of nonselective agonists for functional studies in stably transfected CHO-K1 cell lines. (**A**) The CB1 orthosteric binding site (PDB: 6KPG) docked with CP55,940 (magenta) and AM12033 (teal). (**B**) The CB2 receptor (PDB: 6KPF) in complex with CP55,940 (docked; magenta) and AM12033 (teal) (PDB: 6KPF). Corresponding residues selected for site-directed mutagenesis are highlighted at each receptor. Chemical structures of (**C**) CP55,940 and (**D**) AM12033. Agonist-induced inhibition of forskolin-stimulated cAMP accumulation in cells expressing the (**E**) CB1 or (**F**) CB2 receptor with CP55,940 and AM12033. (**G**) CP55,940, and AM12033 in non-transfected CHO-K1 cells. Data are presented as the mean ± SEM of 3 or more experiments. Potencies can be found in Table 1.

Inspired by the single-particle cryo-EM structures for CB1 (PDB: 6KPG) and CB2 (PDB: 6KPF) [28], we selected the CB1 residues F170^2.57^, F174^2.61^, F177^2.64^, L193^3.29^, Y275^5.39^,C355^6.47^, F379^7.35^, and S383^7.39^ (Figure 1A) and the corresponding CB2 residues F87^2.57^, F91^2.61^, F94^2.64^ I110^3.29^, Y190^5.39^, C257^6.47^, F281^7.35^, and S285^7.39^ (Figure 1B) for site-directed mutagenesis and subsequent functional characterization. The superscript indicates Ballesteros–Weinstein numbering for GPCRs, where the first number represents the transmembrane helix and the second is the residue’s location relative to the most conserved residue within that transmembrane [40]. These amino acid residues were replaced with an alanine in N-terminally HA-tagged human CB1 and CB2 and transduced via a murine stem cell virus into CHO-K1 cells. Cells that were expressing the receptor were isolated using flow cytometry, and receptor surface expression was confirmed by confocal microscopy using a fluorescent HA antibody (Supplemental Figure S1).

The synthetic cannabinoid agonist CP55,940 (Figure 1C) and AM12033 (Figure 1D) are high-affinity agonists at both CB1 and CB2 when measured by their ability to inhibit forskolin-stimulated cAMP accumulation via G_αi_-mediated inhibition of adenylyl cyclase (Figure 1E,F). In this study, we present the % inhibition of forskolin-stimulated cAMP accumulation normalized to the WT receptor response. In Supplemental Figure S2, we present the % forskolin-stimulated cAMP and data showing that agonists at WT receptors produce a 40–50% decrease in cAMP levels with a maximum agonist effect at ~2-fold. No agonist response was detected in the non-transfected, parental CHO-K1 cells (Figure 1G). These agonists were used to compare the impact of the point mutations on signaling in the cAMP assay to the WT response (Table 1).

### Transmembrane 2.

**Figure 2.**
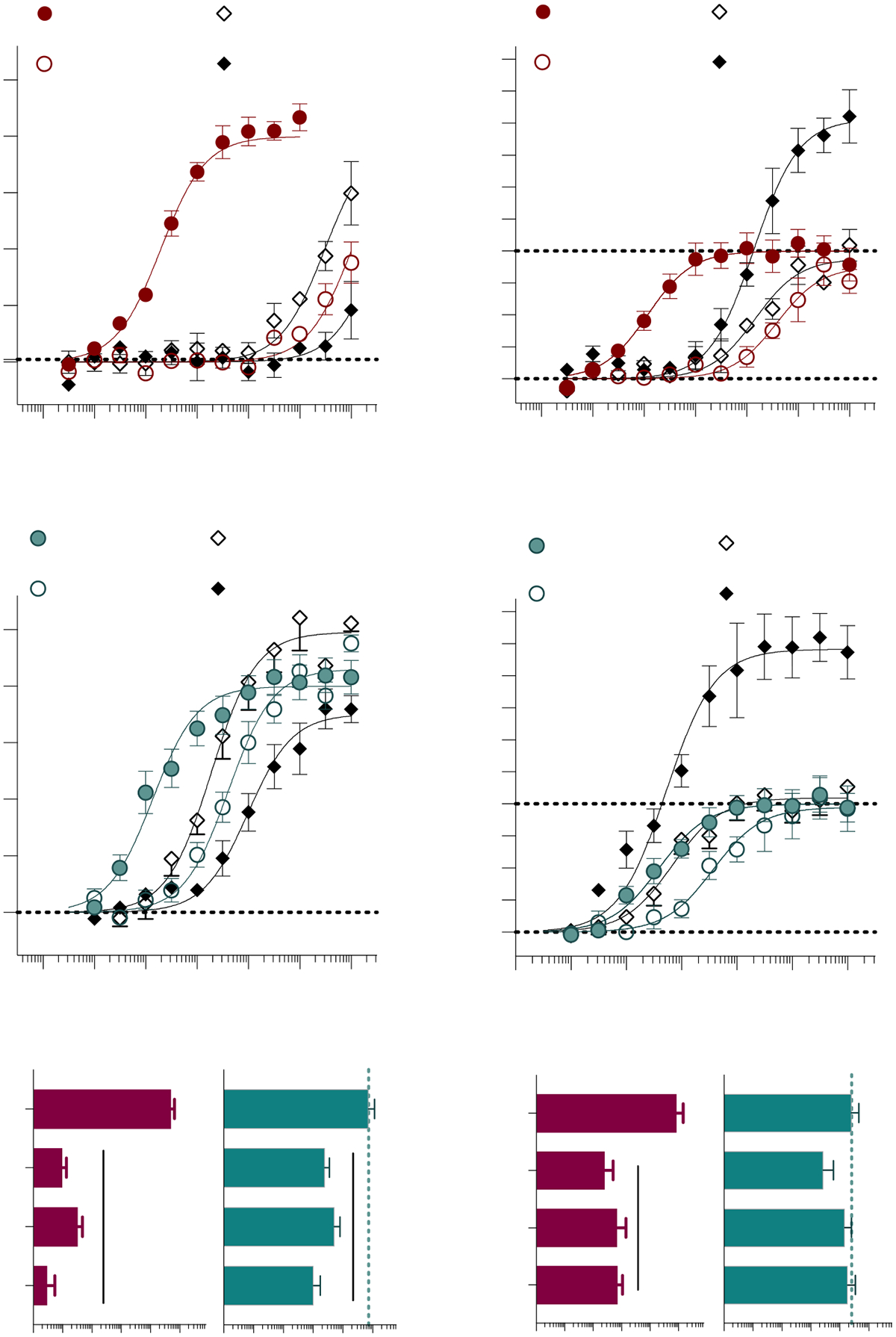
Single-point mutations within Transmembrane 2. Alanine substitutions were made for CB1 at F170A^2.57^, F174A^2.61^, and F177A^2.64^ and hCB2 at F87A^2.57^, F91A^2.61^, and F94A^2.64^ and receptors were stably expressed in CHO-K1 cells. The % inhibition of forskolin-stimulated cAMP accumulation was compared to the WT receptor for CB1 + CP55,940 (**A**); CB1 + AM12033 (**B**); CB1 pEC_50_ summary (**C**); CB2 + CP55,940 (**D**); and CB2 + AM12033 (**E**); CB2 pEC_50_ summary (**F**). Curves are presented with the mean ± SEM of 3 or more experiments run in parallel with WT receptors. The summary of the pEC_50_ values is presented with 95% CI. Statistical comparisons were made to the WT receptor: **** *p* < 0.0001.

The residues selected for mutagenesis in TM2: F170^2.57^, F174^2.61^, and F177^2.64^ at CB1 and F87^2.57^, F91^2.61^, and F94^2.64^ at CB2, contain phenylalanine side chains that engage in aromatic interactions along the orthosteric binding site (for reference, see Figure 1A,B). These mutations dramatically decrease the potency of CP55,940 at both receptors (Figure 2). AM12033 activity is also diminished at CB1; however, at CB2, only the F87A^2.57^ mutation significantly alters the potency of AM12033. Notably, the efficacy of both CP55,940 and AM12033 is nearly doubled in the CB2 F94A^2.64^ mutant. In the WT CB2, CP55,940 and AM12033 decrease forskolin-stimulated cAMP levels by ~40% (% cAMP remaining: CP: 61.3 ± 1.01; AM12033: 62.9 ± 1.1% SEM), while F94A^2.64^ decreases cAMP levels by ~60% (% cAMP remaining: CP: 43.7 ± 2.2; AM12033: 44.2 ± 1.4% SEM) (see Supplemental Figure S2A for data expressed as % cAMP remaining). This increase in efficacy could not be explained by a more robust forskolin response, as stimulated cAMP levels did not differ between WT and the mutant when run in parallel experiments (Supplemental Figure S2B). Notably, this elevation in efficacy was not observed in the corresponding CB1 mutant (F177A^2.64^).

### Transmembranes 3 and 5.

**Figure 3.**
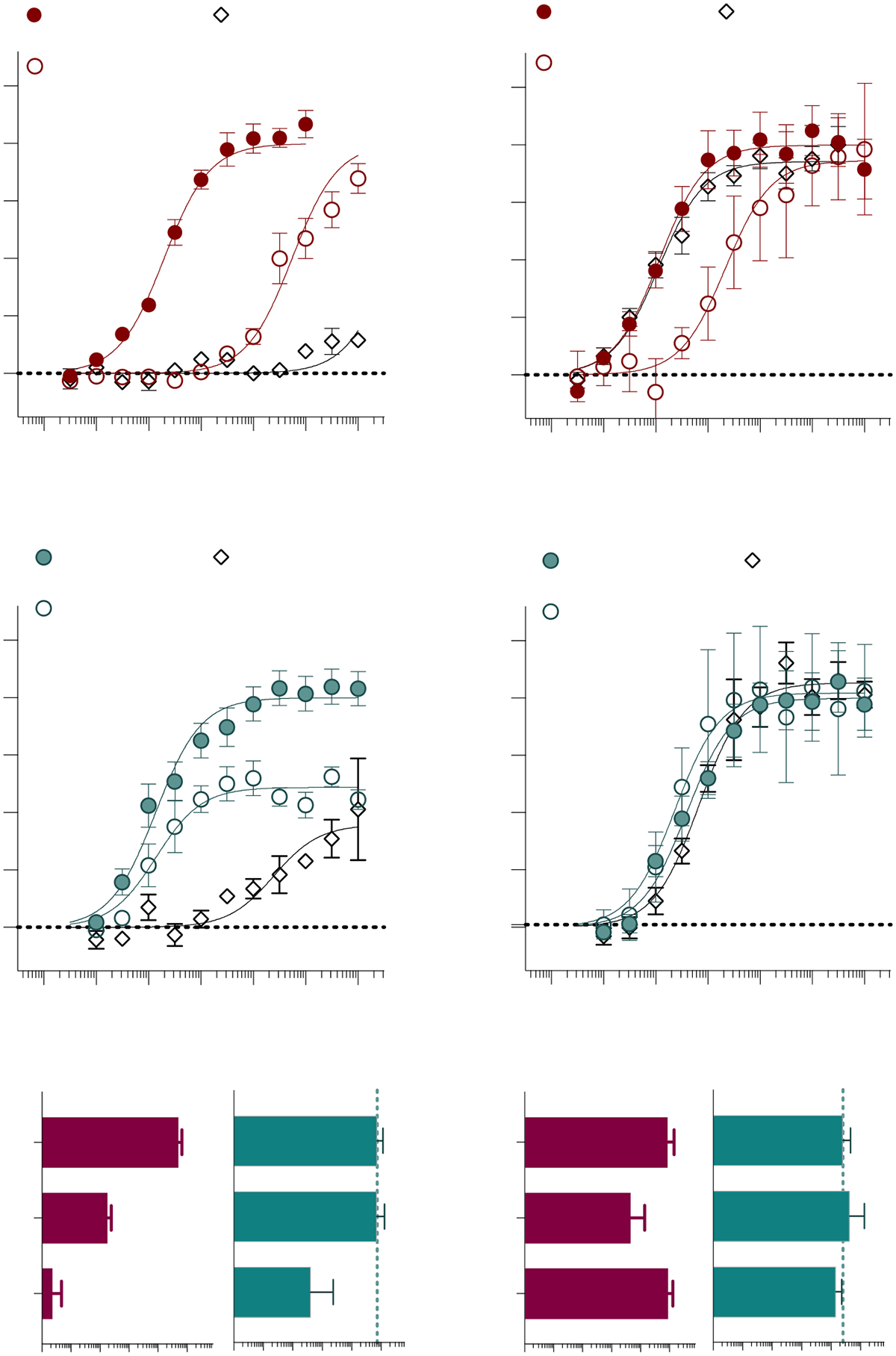
Single-point mutations within Transmembranes 3 and 5. Alanine substitutions were made for CB1 at L193A^3.29^ and Y275A^5.39^ and hCB2 at I110A^3.29^ and Y190A^5.39^ and receptors were stably expressed in CHO-K1 cells. Inhibition of forskolin-stimulated cAMP accumulation was compared to WT receptor for: CB1+ CP55,940 (**A**); CB1 + AM12033 (**B**); CB1 pEC_50_ summary (**C**); CB2 + CP55,940 (**D**); and CB2 + AM12033 (**E**); CB2 pEC_50_ summary (**F**). Curves are presented with the mean ± SEM of 3 or more experiments run in parallel with WT receptors. The summary of the pEC_50_ values is presented with 95% CI. Statistical comparisons were made to the WT receptor: **** *p* < 0.0001, *** *p* < 0.001.

In TM3, leucine (CB1) or isoleucine (CB2) at the 3.29 position have been shown to interact with the aliphatic tail of cannabinoids and influence cannabinoid receptor selectivity [26,28,41]. Both mutations (CB1 L193A^3.29^ and CB2 I110A^3.29^) reduce the potency of CP55,940, while the potency of AM12033 is unaffected (Figure 3).

The Y5.39 position, when mutated to isoleucine, has previously been reported as critical for ligand recognition in both CB1 and CB2 via radioligand binding and functional cAMP inhibition assays [42]. Therefore, it was anticipated that the mutations CB1 Y275A^5.39^ and CB2 Y190A^5.39^ (Figure 3) would disrupt ligand-mediated receptor signaling. Interestingly, the CB1 Y275A^5.39^ mutation decreases the potencies of both CP55,940 and AM12033, while the corresponding residue in CB2 (Y190A^5.39^) has no impact on the potency of either agonist.

**Figure 4.**
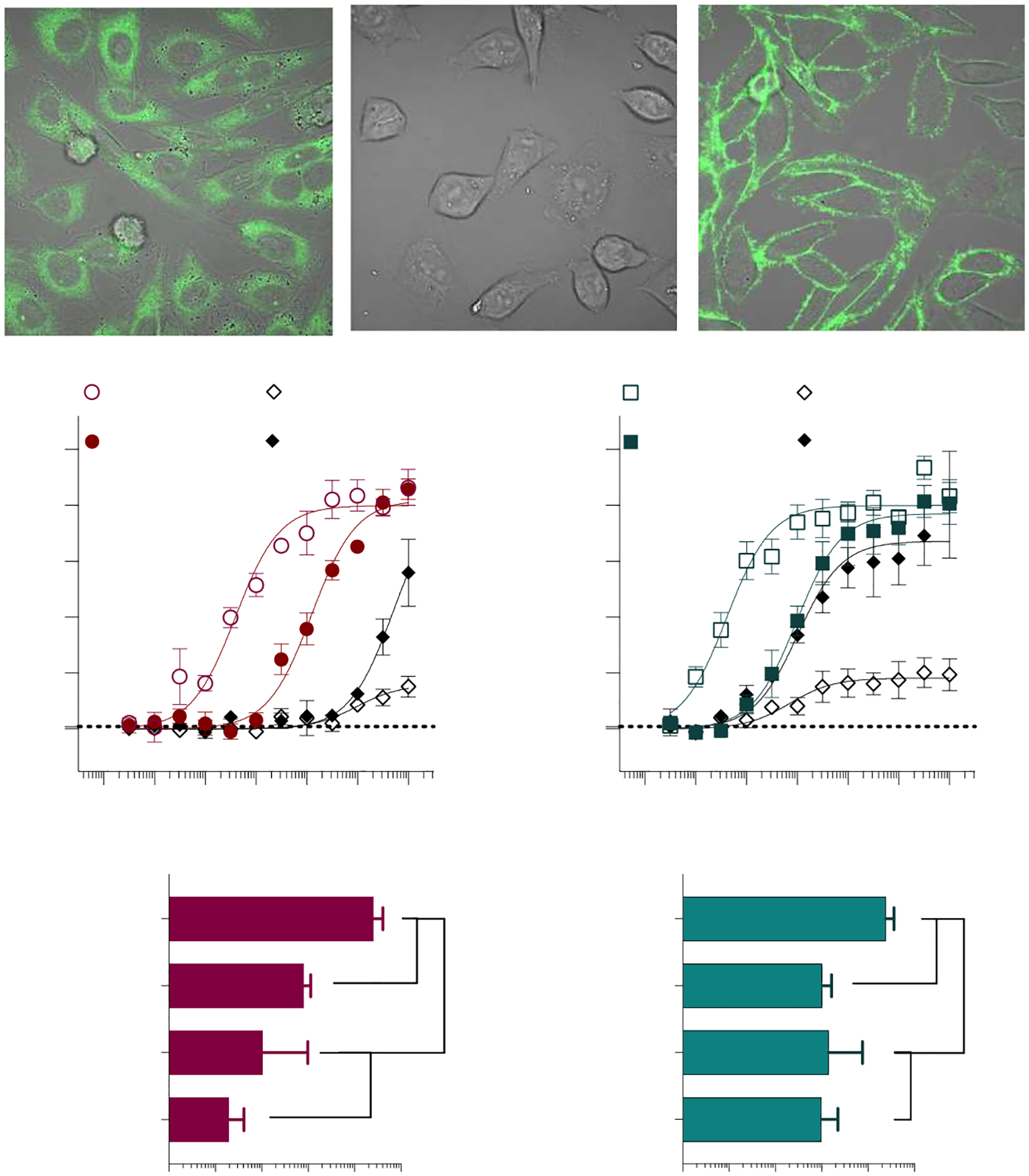
Constitutive internalization of the hCB1 Y275A^5.39^ mutant. Confocal microscopy detects intracellular labeling of the CB1 Y275A^5.39^ mutant stably expressed in CHO-K1 cells upon fixation and permeabilization (**A**) with a fluorescent 488-anti-HA antibody that recognizes the HA tag on the N-terminus of the receptor. No cell surface labeling of the receptor is seen in live cells (**B**). Overnight treatment with the antagonist SR141716A leads to detection of the receptor at the cell surface in live cells (**C**). CP55,940 (**D**) and AM12033 (**E**) activity in the cAMP assay is compared following overnight 10 μM antagonist or vehicle treatment of the wildtype hCB1 and hCB1 Y275A^5.39^. Summaries of the pEC_50_ values for CP55,940 (**F**) and AM12033 (**G**) are presented with 95% CI (**** *p* < 0.0001; ** *p* < 0.01; ns = not significant). Confocal microscopy images were captured with a 100× objective, with a light field applied and no digital zoom; data are representative of more than 3 individual experiments. Curves are presented as the mean of 3 or more individual experiments with S.E.M. See Supplemental Figure S3 for replicates.

Closer examination of the agonist activity curves at CB1 Y275A^5.39^ reveals the possibility of a decrease in efficacy, with underestimated potency (Figure 3A,B). Upon reexamination of the cell line, we found that surface-level expression of CB1 Y275A^5.39^ is variable, and often fleeting upon passaging the stable cell line. Cell fixation and permeabilization mean that HA antibody staining is visible intracellularly under confocal microscopy (Figure 4A, Supplemental Figure S3). However, during live cell imaging, HA antibody staining is not detected (Figure 4B), suggesting that the Y275A^5.39^ CB1 does not make it to the surface or is constitutively internalized. Following treatment with the CB1 antagonist SR141716A [43,44], which is known to halt receptor internalization [45,46], restoration of HA labeling can be observed (Figure 4C). Using this same paradigm, we again assessed agonist activity at the CB1 Y275A^5.39^ mutant following an overnight incubation with the antagonist or vehicle (Figure 4D–F). For the WT CB1 receptor, an ~10-fold decrease in potency for both CP55,940 and AM12033 occurs following the overnight treatment. For the CB1 Y275A^5.39^ mutant, both agonists produce an increase in efficacy upon SR141716A treatment compared to CB1 WT, although no significant change in potency occurs.

### Transmembranes 6 and 7.

**Figure 5.**
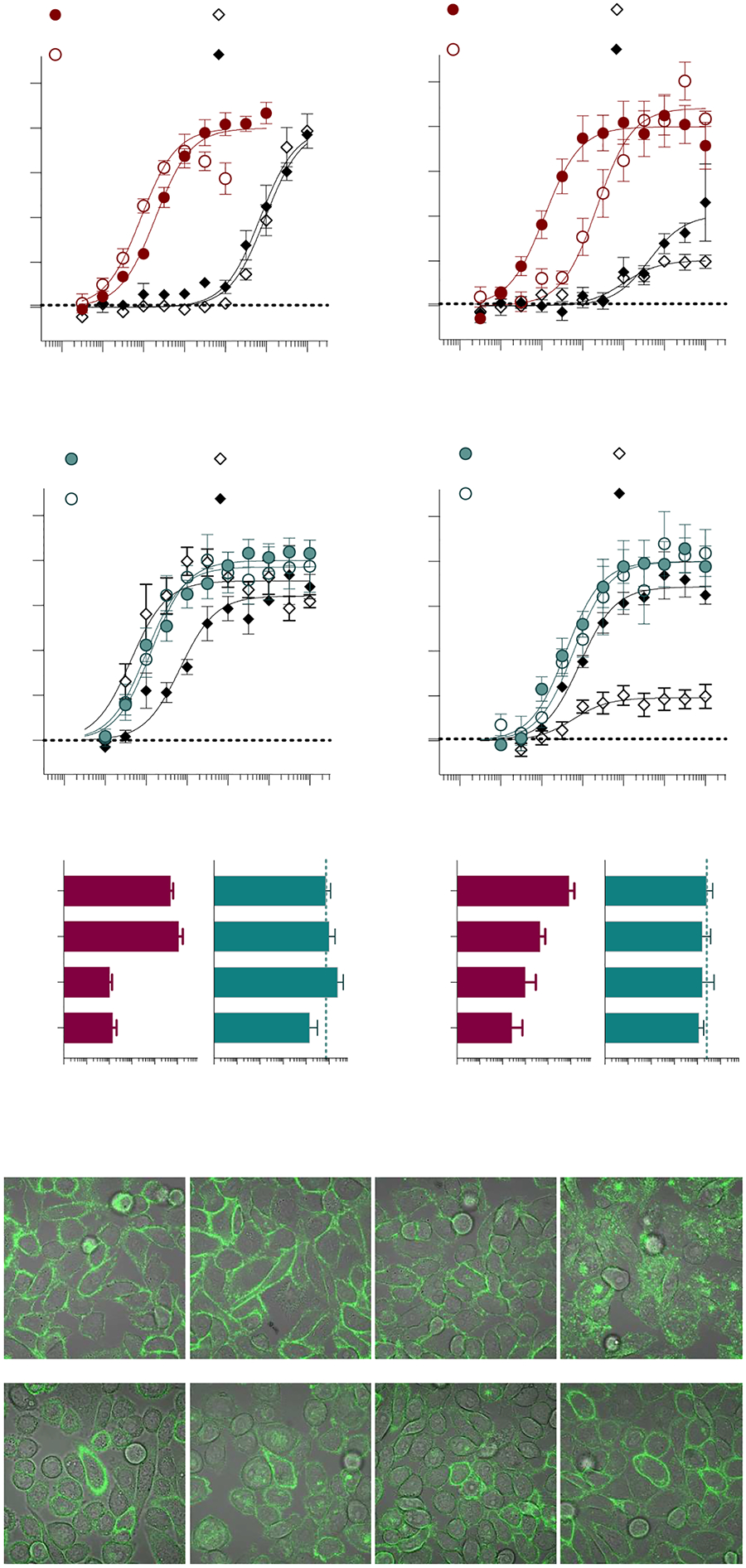
Single-point mutations within Transmembranes 6 and 7. Alanine substitutions were made for CB1 at C355A^6.47^, F379A^7.35^, and S383A^7.39^ and hCB2 at C257A^6.47^, F281A^7.35^, and S285A^7.39^ and receptors were stably expressed in CHO-K1 cells. Inhibition of forskolin-stimulated cAMP accumulation was compared to the WT receptor for: CB1 + CP55,940 (**A**); CB1 + AM12033 (**B**); CB1 pEC_50_ summary (**C**); CB2 + CP55,940 (**D**); and CB2 + AM12033 (**E**); CB2 pEC_50_ summary (**F**). Curves are presented with the mean ± SEM of 3 or more experiments run in parallel with WT receptors. The summary of the pEC_50_ values is presented with 95% CI. Statistical comparisons were made to the WT receptor: **** *p* < 0.0001, *** *p* < 0.001, ** *p* < 0.01. Confocal microscopy of live cells expressing N-terminally tagged CB1 F379A^7.35^ (**G**) or CB2 F281A^7.35^ (**H**) stained with a 488 labeled anti-HA antibody at baseline and following 90 min incubation with the vehicle (media), CP55,940 or AM12033 as indicated in the figure. A loss of membrane definition is only seen with AM12033 in the CB1 mutant, indicative of receptor internalization. Images (100× objective, 2× digital zoom, imaged with fluorescence and transmitted light) are representative of several images taken from 3 independent experiments. See Supplemental Figure S4 for replicates.

The cysteine at position 6.47 is part of the CWxP motif, which is highly conserved among Class A GPCRs [31,47,48]. In CB1 and CB2, C6.47 has been shown to interact with the C3 alkyl chain of various cannabinoids [49–51]. Interestingly, the CB2 C257A^6.47^ mutation decreases the potency of CP55,940, while CP55,940 gains potency in the CB1 C355A^6.47^ mutant. The potency of AM12033 did not significantly differ from the potency in the wild type for either receptor mutated at C6.47 (Figure 5).

The S7.39 position in CB2 has been shown to form a hydrogen bond with the phenolic hydroxyl of AM12033 at C1 [28]. Additionally, the CB2 S285A^7.39^ mutation has also been shown to decrease the potency of CP55,940 in GTPγS binding assays [29] and disrupt CP55,940 radioligand binding [49,52]. We also found this to be true for CP55,940 when measuring the inhibition of cAMP; however, the potency of AM12033 was not significantly affected (Figure 5). The CB1 S383A^7.39^ mutation decreases the potency of both CP55,940 and AM12033 compared to WT (Figure 5).

The F7.35 position is predicted to stabilize the ring system of certain cannabinoids [26]. The potency of CP55,940 is decreased in CB1 F379A^7.35^ relative to WT CB1; however, the potency of AM12033 is significantly improved compared to WT CB1 (Figure 5 A–C). Interestingly, using HA antibody labeling in live cells, we find that CP55,940-induced internalization of CB1 F379A^7.35^ is impaired, while AM12033 leads to robust internalization (Figure 5G, Supplemental Figure S4). The corresponding mutation in CB2, F281A^7.35^, results in a decrease in CP55,940 potency, but no change in AM12033 potency compared to WT CB2 (Figure 5E,F). For both ligands, the maximal stimulation reaches a plateau for CB2F281A^7.35^, but the Emax is significantly lower than observed for the WT CB2 (CP55,940 and AM12033: *p*<0.0001 vs. WT CB2). Cell surface expression of CB2 F281A^7.35^ is comparable to WT CB2, as determined by anti-HA antibody labeling of live cells; however, treatment with either CP55,940 or AM12033 fails to internalize the receptor (Figure 5H).

## 4. Discussion

In this study, we compared the activity of two high-affinity, nonselective cannabinoid agonists for their ability to stimulate inhibitory G protein signaling in CB1 and CB2 receptors with conserved orthosteric site mutations. While many of the mutations decreased the potency of both agonists, there was generally a greater loss in potency for CP55,940 than AM12033 (~two-log-order greater loss for CP55,940 than AM12033 for each receptor (Table 1)). This is likely, in part, due to the bicyclic structure of CP55,940, which allows for more free rotation around the terpene ring and produces less-stable interactions compared to the fixed, tricyclic system of AM12033. Furthermore AM12033 is a log-order more potent than CP55,940 at both receptors in adenylyl cyclase inhibition (AM12033: 0.12 nM at CB1 and 0.38 nM at CB2 and CP55,940: 2.2 nM at CB1 and 1.1 nM at CB2), which is likely attributable to its slow dissociation rate imparted by the cyano moiety on the aliphatic tail [28], thus resulting in high affinity. Taken together, it is not unexpected that single point mutations would have less of an impact on disrupting the activity of the more potent agonist. However, the mutations that affected AM12033 potency the most tended to be at CB1 relative to CB2, providing some evidence for possible subtype selectivity between the receptors.

In the second transmembrane, mutating F2.57, F2.61, or F2.64 to alanine decreases the potency of both CP55,940 and AM12033 at CB1 (Figure 2). This supports earlier radioligand binding studies with the Δ^9^-THC derivative, HU210, and CP55,940 where CB1 residues F174^2.61^ and F177^2.64^ were critical for ligand binding, with F177A^2.64^ being more deleterious than F174A^2.61^, in competition studies against ^3^H-SR141716A [53]. For CB2, there is a significant loss in potency for CP55,940; but only the CB2 F87A^2.57^ mutation impacts AM12033 potency. The decrease in potency of CP55,940 but not AM12033 at CB2F91A^2.61^ and F94A^2.64^, may be due to the high affinity of AM12033 or a result of polar and/or hydrogen bonding interactions between AM12033 and a residue/s that has not yet been identified.

Of note, CB2 F94A^2.64^ shows a 50% increase in efficacy relative to CB2 WT, as was observed with both agonists, while only CP55,940 lost potency. It is difficult to compare the relative efficacy between cell lines as differences in receptor expression levels could impact signaling potency. For example, if a full agonist is to produce a maximal effect, it must occupy some fraction of the total receptor population. If the receptor number increases, the system would be expected to require less agonist to reach the 50% (decrease EC_50_) activation level; thus, it would be expected that the potency would shift leftward as a function of efficacy [54]. However, for AM12033, the potency does not significantly differ from WT, suggesting that F2.64 causes selective efficacy enhancement without a concomitant enhancement of affinity.

TM3, TM4, and TM5 form an aromatic cluster that is important for the binding and signaling of various cannabinoids [42,55–57]. Mutation of Y5.39 to isoleucine in both CB1 and CB2 was shown to abolish ^3^H-CP55,940 binding to both receptors and result in a loss of CP55,940 activity [42]. Therefore, we anticipated the CB1 Y275A^5.39^ and CB2 Y190A^5.39^ mutations would greatly impact the potency of CP55,940 in cAMP inhibition. This hypothesis was supported for CB1, where CP55,940 potency was right shifted more than four log-orders (Figure 3). However, CB2 Y190A^5.39^ had no effect on the potency of CP55,940. These differences in our observations could be due to the difference in sidechain size between isoleucine and alanine, or due to differences in receptor expression between this and the prior study [42]. When docking AM12033 in CB1, we observe the cyano moiety interacting with Y275^5.39^ (as well as T197^3.33^ andI271^ECL2^), while in the CB2 Cryo-EM structure, the cyano moiety is oriented away from the Y5.39 position and interacting with I110^3.29^, F183^ECL2^, and L191^5.40^. AM12033 potency was greatly impacted at CB1 with an approximate two-log-order decrease in potency, while its potency at CB2 was not significantly affected. Therefore, Y5.39 may be a key residue for engaging CB1 but may not be as important for CB2.

Prior studies reported that a CB1 Y275I^5.39^ mutation leads to impaired radioligand binding; intracellular staining of the mutant receptor was also observed [42]. Moreover, we found surface expression of the CB1 Y275A^5.39^ receptor to be inconsistent, yet surface expression could be restored if cultured in the presence of an antagonist (Figure 4). These observations suggest that the Y275A^5.39^ mutation leads to a constitutively internalizing CB1 receptor [58] although it does not eliminate the possibility that the antagonist could serve as a pharmacological chaperone. Restoring surface expression of the Y275A^5.39^ mutant via antagonist treatment preserved the loss of agonist potency; however, overnight antagonist treatment of CB1 WT also decreased agonist potency. The decrease in potency observed for CB1 WT would be consistent with a residual presence of 100 nM SR141716A based on Schild analysis [38], and given that the cells were not washed (to prevent resensitization), it is likely that residual antagonist remained in the system. When the Y275A^5.39^ CB1 mutant was treated in the same manner, the response window was increased, yet no further significant rightward shift in potency was observed for either ligand. Therefore, it is likely that constitutive internalization contributes in a minor way to the loss of agonist potency for the Y275A^5.39^ CB1 receptor, whereas the biggest effects on potency likely reside in the loss of ligand affinity. That is, mobilization of the Y275A^5.39^ mutant to the surface does not significantly increase agonist potency as would be expected for a high-affinity ligand; agonist potency would not be expected to be to the right of agonist affinity. Several Class A GPCRs have a phenylalanine or tyrosine residue at position 5.38 or 5.39, and in some cases, mutating this position to alanine impairs receptor internalization [59]. CB1, however, internalizes regardless of the mutation of this residue.

Cannabinoid entry into the orthosteric pocket is proposed to occur through the lipid bilayer [60,61]; with this in mind, interactions between the CWxP motif and cannabinoid ligands are possible. Studies evaluating C355A^6.47^ in CB1 showed no effect on ^3^H-CP55,940 affinity [50]; nor was CP55,940 signaling affected in GTPγS binding assays [62]. In the cAMP assay, we observed a significant increase in the potency of CP55,940 at CB1 C355A^6.47^ with no effect on AM12033. Interestingly, for CB2 C257A^6.47^, the potency of CP55,940 decreased by approximately one log-order, while AM12033 was unaffected (Figure 5). This suggests that this residue may be important for conferring agonist activity at CB2; however, the cause of the difference in activity remains unknown. Due to the residue’s proximity to a proposed cannabinoid entry site, it is possible that this mutation affects ligand entry at CB2.

The F7.35 position is thought to stabilize the ring system of the cannabinoid structure through hydrophobic interactions, and mutation to alanine for CB1 results in a loss of potency for CP55,940 in cAMP inhibition [26]. We observed that CP55,940 loses potency at both CB1 F397A^7.35^ and CB2 F281A^7.35^ (Figure 5). However, the potency of AM12033 is improved in CB1 F379A^7.35^ and unchanged in F281A^7.35^; meanwhile, efficacy is dramatically reduced for both agonists at CB2. Both CB1 and CB2 are known to internalize in response to agonist treatment [45,46,63–66]. While surface labeling was intact for the F7.35A mutants, only AM12033 promoted internalization at CB1 F397A^7.35^, while the CB2 F281A^7.35^ receptor did not internalize in response to either agonist treatment (Figure 5G,H). GPCRs are known to promote G protein signaling from endosomes [67], and both CB1 and CB2 have been proposed to signal in endosomes [68–70]. It is possible that the inability of F281A^7.35^ to internalize could lead to the decreased efficacy of both ligands; however, the role of endocytosis for CB2 G_αi_-mediated inhibition of adenylyl cyclase (i.e., cAMP accumulation) has not been explored. Regardless, since CB2 F281A^7.35^ fails to efficiently internalize upon agonist stimulation, it may be key for understanding interactions that determine functional selectivity at CB2. Additional studies are underway to further improve our understanding of this relationship between agonist occupancy and receptor signaling in the CB2 F281A^7.35^.

## 5. Conclusions

As predicted from the captured structures and the agonist docking on these structures, mutations that likely disrupt agonist engagement also disrupt agonist-induced activation of G protein signaling. While the effects on signaling support the docking predictions, we acknowledge that the mutations could have wider-reaching effects on receptor conformation that could also impact receptor signaling to G proteins. Indeed, substitution of aromatic side chains and polar groups can indirectly affect the receptor ensemble, and subsequently, decrease the efficiency of signal transduction. Moreover, additional residues, which are not obvious from the ligand binding poses, may still serve as essential residues that facilitate the ultimate best pose of an agonist in the pocket [30–33]. Subsequent investigations, involving additional sites gleaned from more refined molecular dynamic and structural studies, alone and in concert with known mutations will help to address these questions. From this study, the most pivotal positions identified are C6.47, which enhances the activity of CB1 yet decreases the activity of CB2, and Y5.39, which inhibits CB1 activity yet has no effect on CB2. The mutation of Y275^5.39^ to alanine leads to constitutive internalization of CB1, and F281A^7.35^ decreases the internalization of CB2, which appears to effect efficacy in the cyclase assay. Additional studies investigating other downstream signaling pathways, including internalization, βarrestin recruitment, and ERK signaling, may provide greater insight into how ligands confer functional selectivity on cannabinoid receptors. Taken together, our findings add to the rapidly growing literature, which may help ultimately inspire the future structure-guided drug design of more selective agonists and antagonists at CB1 and CB2.

## Supplementary Material

sup fig

## Figures and Tables

**Table 1.** CP55,940 and AM12033 potencies in WT and mutant CB1 and CB2 in the forskolin-stimulated cAMP accumulation assay (HTRF) in stably transfected CHO-K1 cells.

	CP55,940			AM12033		
CB1	EC_50_, nM	(95% CI)	vs. CB1	EC_50_, nM	(95% CI)	vs. CB1
WT CB1	1.97	(1.52–2.54)		0.137	(0.0853–0.219)	
F170A^2.57^	10368	(7440–14779)	****	4.07	(2.82–5.86)	****
F174A^2.61^	3072	(2126–4427	****	1.90	(1.24–2.89)	****
F177A^2.64^	>10,000		****	9.76	(5.75–16.3)	****
L193A^3.29^	549	(405–744)	****	0.139	(0.0761–0.247	
Y275A^5.39^	>10,000		****	25	(4–130)	***
C355A^6.47^	0.870	(0.576–1.315)	**	0.100	(0.0553–0.177)	
F379A^7.35^	946	(760–1173)	****	0.042	(0.0241–0.0718)	**
S383A^7.39^	713	(471–1075)	****	0.685	(0.322–1.39)	**
	CP55,940			AM12033		
CB2	EC_50_, nM	(95% CI)	vs. CB2	EC_50_, nM	(95% CI)	vs. CB2
WT CB2	1.17	(0.681–1.99)		0.393	(0.217–0.705)	
F87A^2.57^	388	(198–735)	****	3.71	(1.65–8.12)	****
F91A^2.61^	146	(71–282)	****	0.686	(0.389–1.19	
F94A^2.64^	139	(93–207)	****	0.532	(0.289–0.944)	
I110A^3.29^	22	(7–64)	****	0.233	(0.073–0.698)	
Y190A^5.39^	1.13	(0.770–1.65)		0.679	(0.437–1.04)	
C257A^6.47^	23	(13–38)	***	0.606	(0.264–1.36)	
F281A^7.35^	100	(34–269)	****	0.586	(0.189–1.76)	
S285A^7.39^	424	(147–1160)	****	0.856	(0.538–1.35)	

**** *p* < 0.0001;

*** *p* < 0.001,

** *p* < 0.01.

## Data Availability

Data have been graphed and presented in the manuscript, raw values are available upon request.
