# Supplementary material for: Comparison of Agonist Activity between CB1 and CB2 Receptors with Orthosteric Site Mutations
Source: Receptors (Basel). Author manuscript; Available in PMC 2025 May 15. (PMC12080992; doi:10.3390/receptors3030018)
Supplement: sup fig [file NIHMS2067872-supplement-sup_fig.pdf]

# Table S1. Primers

| | | | Forward | Reverse |
|---|---|---|---|---|
| **CB1** | F170A | Sigma | 5’ – GTGTCATTGCTGTCTACAGC – 3’ | 5’ – TCCCCAGGAGGTCTG – 3’ |
| | F174A | Sigma | 5’ – CTACAGCGCCATTGACTTC – 3’ | 5’ – ACAAAAATGACACTCCCCAG – 3’ |
| | F177A | Sigma | 5’ – CTTCATTGACGCCCACGTGTT – 3’ | 5’ – CTGTAGACAAAAATGACACTCCCC – 3’ |
| | L193A | Sigma | 5’ – TCTGTTCAAAGCGGGTGGGGT – 3’ | 5’ – AACACGTTGCGGCTATCTTTGC – 3’ |
| | Y275A | Sigma | 5’ – GATGAAACCGCCCTGATGTTC – 3’ | 5’ – AATGTGTGGGAAAATGTCTG – 3’ |
| | C355A | Sigma | 5’ – GTTGATCATCGCCTGGGGCCCTCTG – 3’ | 5’ – ACCACCAGGATCAGGACC – 3’ |
| | S383A | Sigma | 5’ – TGCGCTATGCTCTGCCT– 3’ | 5’ – GAATGCAAACACCGTCTTAATGAGC – 3’ |
| | F379A | Sigma | 5’ – GTGGCTGCATTCTGC – 3’ | 5’ – CGTCTTAATGAGCTTGTTCATC – 3’ |
| **CB2** | F87A | Sigma | 5’ – GTCGCTGCATGCAGC– 3’ | 5’ – CACACTGGCCAGGAAGTC – 3’ |
| | F91A | Sigma | 5’ – CAGCGCTGTGAATTTCCATG– 3’ | 5’ – CATGCAAAGACCACACTGGC – 3’ |
| | F94A | Sigma | 5’ – GAATGCCCATGTTTTCCATGG – 3’ | 5’ – ACAAAGCTGCATGCAAAGACC– 3’ |
| | I110A | GeneScript | | |
| | Y190A | Sigma | 5’ – GACGCCCTGCTGAGCT – 3’ | 5’ – ATTGGGGATCAGTGGGAAAAG – 3’ |
| | C257A | Sigma | 5’ – GCTCCTCATCgcTTGGTTCCCAGTGCTG – 3’ | 5’ – ACAGCCAACACTAGCCCT – 3’ |
| | F281A | GeneScript | | |
| | S285A | GeneScript | | |

**Supplemental Figure S1.**

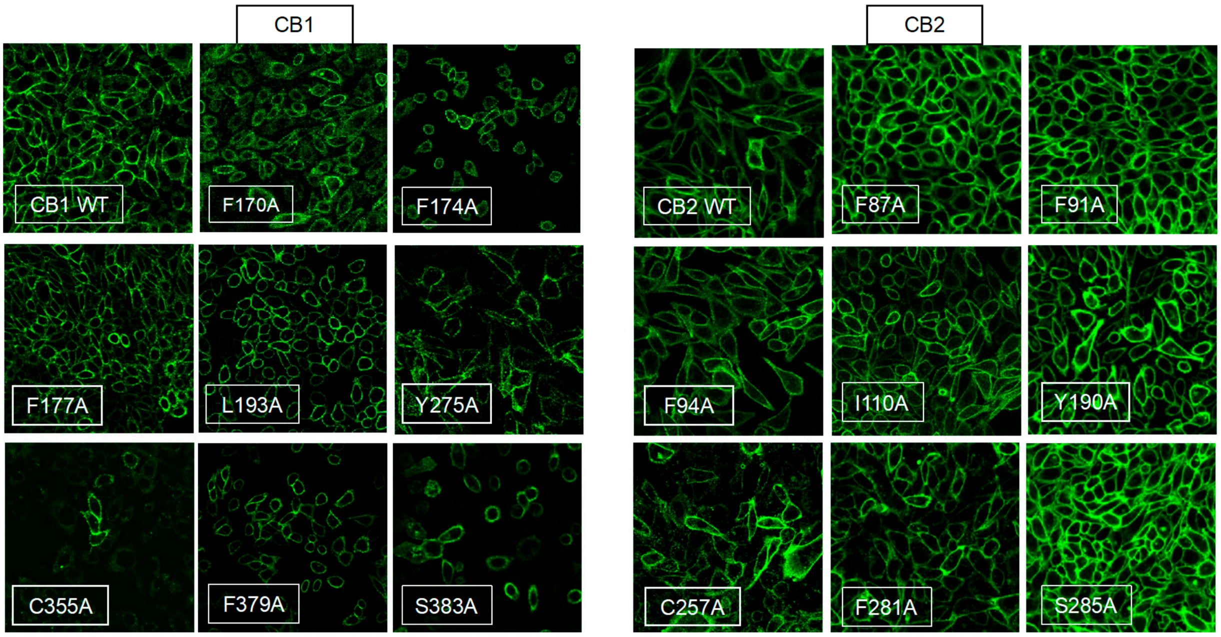


**Supplemental Figure S1. Nonpermeabilized CHO-K1 cells stained with DyLight 488 HA-antibody.** Confocal images were taken at 40x or 60x and were imaged following flow cytometry isolation of surface receptor expressing cells (N-terminally HA tagged receptors).

## Supplemental Figure S2.

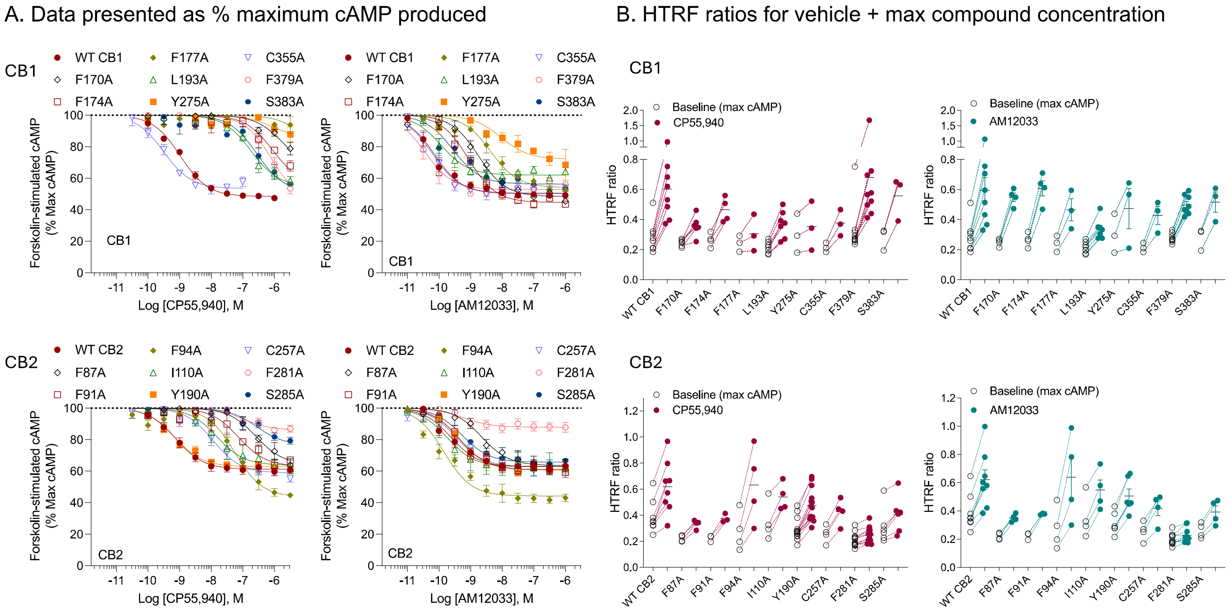


**Supplemental Figure S2 to accompany Figures 1-3 and 5.** Data in the manuscript presented as **A).** % of maximal cAMP stimulated in the transfected CHO-K1 cells (mean with SEM). For the WT CB1, CP55,940 and AM12033 decrease forskolin-stimulated cAMP levels by ~50% (%cAMP remaining: CP: 48.4 ± 0.9; AM12033: 50.2 ± 1.0 % mean with SEM). For the WT CB2, CP55,940 and AM12033 decrease forskolin-stimulated cAMP levels by ~40 % (% cAMP remaining: CP: 61.3 ± 1.01; AM12033: 62.9 ± 1.1% mean with SEM); the F94A2.64 decreases cAMP levels by ~60% (% cAMP remaining: CP: 43.7 ± 2.2; AM12033: 44.2 ± 1.4 % mean with SEM, three parameter nonlinear regression with top constrained to 100%, GraphPad Prism). **B).** Raw HTRF ratios showing the values of vehicle treatment (maximum forskolin stimulated cAMP levels) and the maximum dose tested for each compound in (A). Ordinary one-way ANOVA comparing Baselines (max cAMP) between WT and each mutant indicate that for CB1, none of the mutants differed from WT. For CB2, F87A ($p<0.01$) and F281A ($p<0.001$) had higher forskolin-stimulated cAMP levels than WT (lower HTRF values).

**Supplemental Figure S3.**

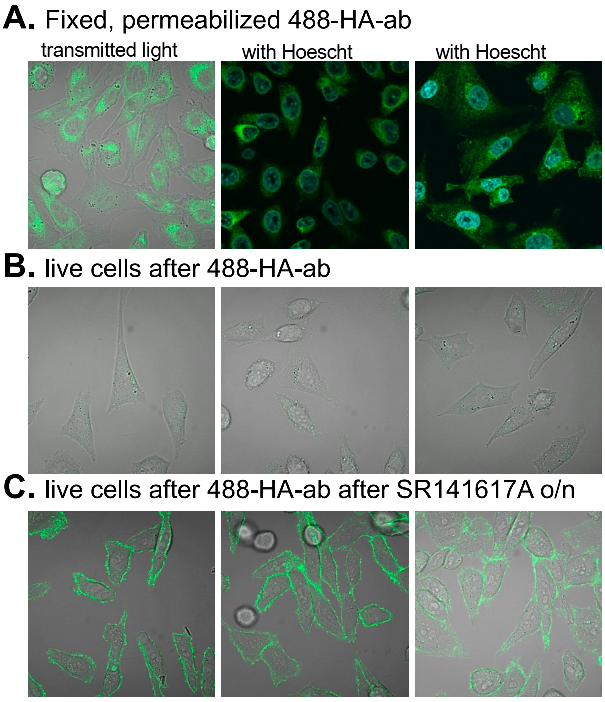


**Supplemental Figure S3 to accompany Figure 4.** CHO-K1 cell surface expression profile of N-terminally tagged HA-CB1 Y275A$^{5.39}$ in representative images of 3 independent studies. After several passages, 488-HA antibody was observed only upon fixation and permeabilization of cells (**A**) shown with transmitted light and following Hoescht staining of nuclei. No cell surface staining of live cells were observed with the 488-HA antibody (**B**). Overnight treatment with 10 µM SR141617A restores surface labeling of HA-CB1 Y275A$^{5.39}$ in live cells (**C**).

## Supplemental Figure S4.

**A.**

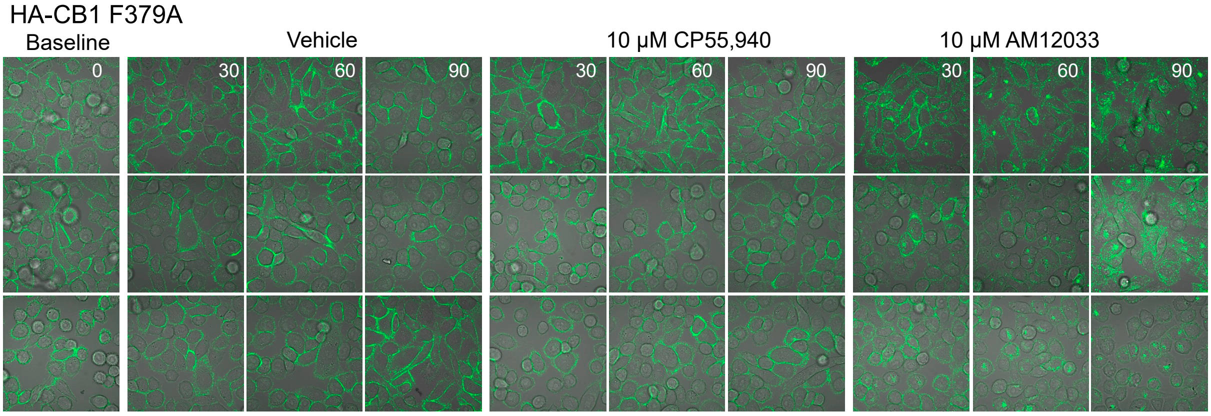


**B.**

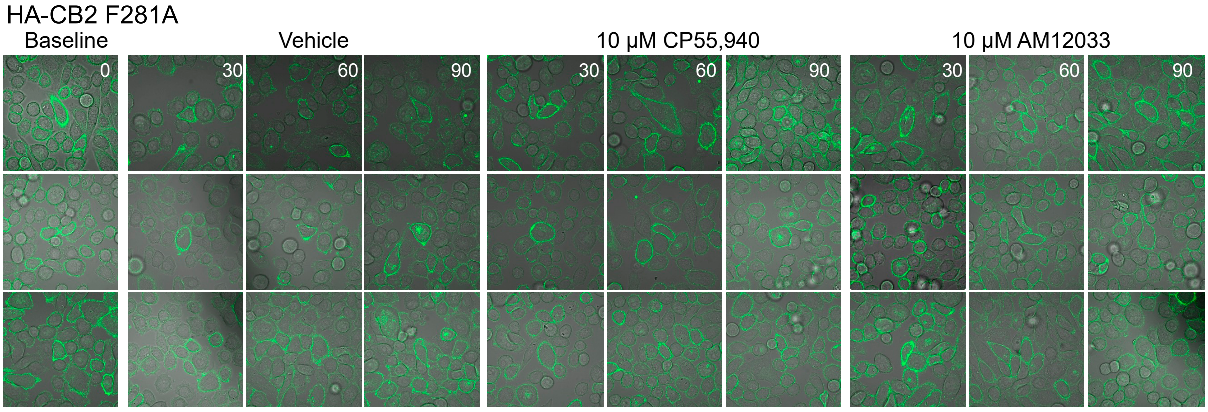


**Supplemental Figure S4 to accompany Figure 5.** Internalization profiles of the N-terminally tagged F7.35A mutant CB receptors in response to Vehicle, 10 µM CP55940 or 10 µM AM12033 over time in live CHO-K1 cells. Cell surface labeling is evident under all conditions for (**A**) CB1 F379A$^{7.35}$ while AM12033 leads to a loss of surface labeling for (**B**) CB2 F281A$^{7.35}$. Baseline images were taken after fluorescent 488-HA antibody staining of live cells and prior to addition of indicated treatment. Replicate data from 3 separate transfections representing baseline, Vehicle, CP55,940 or AM12033 treatment over time (indicated in minutes as 0, 30, 60, or 90 min; 100X objective for all images).
